# Jisuikang Promotes the Repair of Spinal Cord Injury in Rats by Regulating NgR/RhoA/ROCK Signal Pathway

**DOI:** 10.1155/2020/9542359

**Published:** 2020-11-28

**Authors:** Chengjie Wu, Yuxin Zhou, Pengcheng Tu, Guanglu Yang, Suyang Zheng, Yalan Pan, Jie Sun, Yang Guo, Yong Ma

**Affiliations:** ^1^Department of Traumatology and Orthopedics, Affiliated Hospital of Nanjing University of Chinese Medicine, Nanjing, China; ^2^Laboratory of New Techniques of Restoration & Reconstruction, Institute of Traumatology & Orthopedics, Nanjing University of Chinese Medicine, Nanjing, China; ^3^Laboratory of Chinese Medicine Nursing Intervention for Chronic Diseases, Nanjing University of Chinese Medicine, Nanjing, China

## Abstract

Jisuikang (JSK) is an herbal formula composed of many kinds of traditional Chinese medicine, which has been proved to be effective in promoting the rehabilitation of patients with spinal cord injury (SCI) after more than ten years of clinical application. However, the mechanisms of JSK promoting nerve regeneration are yet to be clarified. The aim of this study was to investigate the effects of JSK protecting neurons, specifically the regulation of NgR/RhoA/ROCK signal pathway. The motor function of rats was evaluated by the BBB score and inclined plate test, Golgi staining and transmission electron microscope were used to observe the microstructure of nerve tissue, and fluorescence double-labeling method was used to detect neuronal apoptosis. In this study, we found that JSK could improve the motor function of rats with SCI, protect the microstructure (mitochondria, endoplasmic reticulum, and dendritic spine) of neurons, and reduce the apoptosis rate of neurons in rats with SCI. In addition, JSK could inhibit the expression of Nogo receptor (NgR) in neurons and the NgR/RhoA/ROCK signal pathway in rats with SCI. These results indicated JSK could improve the motor function of rats with SCI by inhibiting the NgR/RhoA/ROCK signal pathway, which suggests the potential applicability of JSK as a nerve regeneration agent.

## 1. Introduction

Spinal cord injury (SCI) is a disease with severe damage to the central nervous system, which has a complex pathological process and is difficult to recover in the later stage. The incidence of SCI is increasing year by year, so it is urgent to find a treatment to reduce the disability rate [1, 2]. However, traditional Chinese medicine has been concerned by more and more clinicians and scholars because of its advantages of multitargets and low side effects. The pathogenesis core of SCI is “deficiency of kidney governor, stasis of governor pulse, and dereliction of duty of cardinal command.” JSK is a compound prescription of traditional Chinese medicine established under the guidance of this theory, which is very effective in the clinical treatment of SCI [3, 4].

In China, Buyang Huanwu decoction, as an important representative herbal formula for the treatment of SCI, has the function of restoring nerve and has a history of hundreds of years [5, 6]. However, the mechanism of Buyang Huanwu decoction is not clear, mainly related to thioredoxin system [7], glutamate [8], cAMP/CREB/RhoA signal pathway [9], apoptosis-related proteins [10], etc. JSK is formed by the addition and subtraction of Buyang Huanwu decoction, which is in line with the current clinical characteristics of SCI. After a large number of clinical tests, JSK has achieved satisfactory clinical efficacy, and part of its mechanism has been explained by animal and cell experiments [3, 4, 11–14]*. Astragalus membranaceus*, *Salvia miltiorrhiza*, and Ligustrazine are the main pharmacological components of JSK and have important therapeutic effects. *Astragalus membranaceus* has antioxidant and neuroprotective effects in vivo, which provides the possibility for improving the symptoms of SCI [15–17]. *Salvia miltiorrhiza* has the effects of promoting blood circulation [18], antioxidation, and anti-inflammation [19], neuroprotection [20], etc. Ligustrazine can protect SCI by inhibiting inflammatory cytokines [21], inhibiting apoptosis [22, 23], and scavenging oxygen free radicals [24].

SCI is generally considered to be a devastating injury, and the growth of axons in the central nervous system is limited, so it could not be recovered. However, Aguayo et al. transplanted part of the peripheral nerve tissue into the transected spinal cord and found that the axons of the central nervous system could grow into the transplanted tissue, suggesting that neurons could grow after the adult central nervous system was damaged, but it was limited by the environment [25, 26]. Other studies have shown that the white matter homogenate of the central nervous system limits the growth of nerve axons [27], and oligodendrocytes and myelin sheath of the central nervous system can induce the collapse of the growth cone [28]. These proteins that inhibit axon growth are called myelin-associated inhibitors (MAIs), which mainly include neurite outgrowth inhibitor (Nogo), myelin-associated glycoprotein (MAG), and oligodendrocyte myelin glycoprotein (OMgp) [29]. Nogo receptor (NgR) can specifically bind to the above three kinds of MAIs and inhibit axonal regeneration [30], and the expression of NgR is directly related to the ability of central nervous cell regeneration [31], so NgR is considered to be one of the important targets for promoting axonal regeneration. In addition, the NgR/RhoA/ROCK signal pathway is an important way to inhibit neuronal axonal regeneration. Therefore, in this study, the development of NgR/RhoA/ROCK signal pathway in rats with SCI in different time periods and the intervention effect of JSK were studied to explore part of the mechanism of improving SCI.

## 2. Methods and Materials

### 2.1. Chemicals

Prednisone (Tianjin Chemical Company, Tianjin, China) was dissolved and diluted in salt solution (final concentration is 0.3%), pentobarbital sodium (China Pharmaceutical Group Shanghai Chemical Reagents Co. LTD), and paraformaldehyde (Nanjing Fumace Biotechnology Co. LTD); JSK was composed of milkvetch root (30 g), Chinese angelica (12 g), red peony root (12 g), earthworm (10 g), *Cistanche deserticola* (10 g), *Salvia miltiorrhiza* (10 g), Szechwan lovage rhizome (10 g), peach seed (10 g), safflower (10 g), etc. (Pharmacy, Affiliated Hospital of Nanjing University of Chinese Medicine). JSK (crude drug 1.25 g/mL) was prepared by boiling, steam boiling, and concentration.

### 2.2. Animals

The animals used in this study were from the Animal Experimental Center of Nanjing University of Chinese Medicine, and the research scheme was approved by the Animal Ethics Committee of the affiliated Hospital of Nanjing University of Chinese Medicine. The experimental procedure follows the National Institutes of Health Guide for the Care and Use of Laboratory Animals (Institute of Laboratory Animal Resources 1996). Eighty female SD rats with weight of 180∼200 g were purchased from Qinglongshan Animal Breeding Farm in Nanjing, and the experimental animal license number was SYXK (SU) 2018-0049. Rats were kept in captivity at the Animal Experimental Institute of Nanjing University of Chinese Medicine (Nanjing, China) to eat and drink freely under controlled temperature and humidity.

### 2.3. SCI Rat Model

The acute SCI model based on the improved Allen method was used [32]. In short, all rats were anesthetized intraperitoneally with 1% pentobarbital sodium (50 mg/kg). The segment T9-11 of spinal cord was exposed surgically, and the segment T10 of spinal cord was hit with spinal cord percussion device (RWD Company). The weight and diameter of the falling object were 10 g and 2.5 mm, respectively, and the falling height and depth were 10 cm and 2 mm, respectively. The tension of the rat hind limbs was eliminated immediately, indicating that the model was established successfully. In the sham group, the spinal cord was exposed but not injured. The rats were randomly divided into four groups: sham group (intragastric infusion with normal saline, 20 mL/kg/d), model group (intragastric infusion with normal saline, 20 mL/kg/d), prednisone (PED) group (intragastric infusion with prednisone, 60 mg/kg/d), and JSK group (intragastric infusion with JSK, 25 g/kg/d). The rats were killed after the last intervention, and the samples were taken on the 7th, 14th, 21^st^, and 28th days after injury.

### 2.4. Basso, Beattie, and Bresnahan (BBB) Score

According to the observation of hindlimb movement, especially gait and coordination, the BBB test was carried out in the open field. Through double-blind and double-independent observation, all groups were evaluated on the 7th, 14th, 21^st^, and 28th days after operation [33].

### 2.5. Oblique Board Test

The oblique board test was carried out on all rats before operation and on the 1st, 7th, 14th, 21^st^, and 28th day after operation. The rats were placed on a rectangular oblique board perpendicular to the longitudinal axis of the oblique board. The oblique board was lifted, and the maximum angle was recorded at which the rats stayed on the board for more than 5 seconds. Each rat was tested for 3 times, and the average value was taken as the final result.

### 2.6. Golgi Staining

The spinal cord was fixed in 4% polyformaldehyde solution and cut into 2-3 mm thick tissue blocks after rinsing. The brain tissue blocks were completely immersed in Golgi staining solution and treated without light for 14 days. After being removed, it was dehydrated at 4°C in 15% sucrose solution and dehydrated for 1 day under the condition of avoiding light and dehydrated in 30% sucrose solution for 2 days. It was immersed in concentrated ammonia water for 45 minutes, distilled water for 1 minute, fixing solution treatment for 45 minutes, and distilled water washing for 1 minute. It was dehydrated in 30% sucrose solution for 2-3 days and cut into 100 *μ*m frozen section, sealed by glycerin gelatin. Microscopic examination and image acquisition were carried out.

### 2.7. Observation by Using Transmission Electron Microscope

After the spinal cord was removed, it was fixed with electron microscope fixation solution at 4°C for 2–4 hours and rinsed with 1% osmic acid at 20°C for 2 hours. The organization in turn enters 50%-70%-80%-90%-95%-100%-100% alcohol-100% acetone-100% acetone, each time by 15 minutes. Acetone: 812 embedding agent = 1 : 1 was permeated for 2–4 hours; acetone: 812 embedding agent = 2 : 1 was permeated overnight, and pure 812 embedding agent was permeated for 5–8 hours, and then it was in 37°C oven overnight and polymerized at 60°C for 48 hours. It was cut into the ultrathin slices of 60–80 nm and double stained by uranium and lead, and the slices were dried at room temperature overnight. The images were collected and analyzed under the transmission electron microscope.

### 2.8. Fluorescent Double-Labeling Method of TUNEL and NeuN

Frozen sections of the spinal cord were fixed with 4% paraformaldehyde for 15 minutes and washed twice by PBS. 0.5% TritonX-100 was added to incubate for 5 minutes at room temperature. 5% fetal bovine serum was added at room temperature for 2 hours; NeuN first antibody of 1 : 1000 was added and incubated overnight at 4°C and washed 3 times. Fluorescent secondary antibody Alexa fluor 594 was added and incubated at room temperature and hidden from light for 1 hour. According to the instructions of the TUNEL kit, proper amount of detection solution was added sequentially and washed 3 times after incubation at 37°C for 1 hour. The plates were sealed with antifluorescence quenching solution and observed under the fluorescence inverted microscope.

### 2.9. Fluorescent Double-Labeling Method of NeuN and NgR

Frozen sections of spinal cord were fixed with 4% paraformaldehyde for 15 minutes, permeated with 0.5% Triton-100 for 10 minutes, and washed with PBS for 3 times. 5% goat serum was added at room temperature for 60 minutes. The first antibody NeuN (1 : 300) and NGR (1 : 300) was added and incubated overnight at 4°C. The second antibody Alexa fluor 594 and Alexa fluor 488 was added and incubated for 1 hour. After Dapi restaining, the plates were observed under fluorescence inverted microscope.

### 2.10. Immunohistochemistry

After the paraffin sections were dewaxed to water, the slides were immersed in citric acid antigen repair buffer, boiled water for antigen repair. They were transferred to 3% hydrogen peroxide solution and incubated in room temperature for 25 minutes. 3% BSA was dripped to cover the tissue evenly at room temperature for 30 minutes. The first antibody was dripped and incubated overnight at 4°C. HRP-labeled secondary antibody was dripped and incubated at room temperature for 60 minutes. After drying, the slices were dripped with DAB chromogenic solution, then hematoxylin restaining was done, followed by hydrochloric acid alcohol differentiation, ammonia water returning to blue, and rinsed with running water. The slices were dehydrated and transparent in gradient alcohol and xylene, and the plates were observed under the microscope.

### 2.11. Western Blot Assay

The protein from the spinal cord was extracted and quantified by the BCA method. After adding buffer, the protein was boiled for 10 minutes and stored at −20°C to be tested. After preparing the glue, electrophoresis was carried out by 100 V constant voltage for 90 minutes, and PVDF membrane was used by 100 V for 60 minutes. 5% skimmed milk powder was added at room temperature for 2 hours, GAPDH and NgR, RhoA, and ROCK antibodies were prepared according to the ratio of 1 : 1000 and incubated overnight at 4°C. On the next day, the second antibody was prepared according to the proportion of 1 : 10000 and incubated at room temperature for 2 hours. An ECL developer was added, a gel imaging system was developed, and the ImageJ image analysis system was used to analyze the bands.

### 2.12. qRT-PCR

The RNA in the spinal cord was extracted with RNAiso for RNA (Takara, Japan) and then reverse transcribed with TaKaRa reverse transcription kit and frozen at −20°C. The sequence was found on Genbank, and primers were designed and synthesized in Shanghai Shenggong. GAPDH and NgR were detected by qPCR using TaKaRa TB Green TM Premix Ex Taq TM II PCR kit (Takara, Japan).

### 2.13. Statistic Analysis

SPSS 20.0 software was used for statistical analysis. The data were shown as mean ± SD. One-way ANOVA and SNK-q test were used to analyze the differences among groups. The figures were edited by GraphPad Prism 8.0.2 software. A value of *P* < 0.05 was considered statistically significant.

## 3. Results

### 3.1. JSK Promotes the Recovery of Motor Function in Rats with SCI

**Figure 1:**
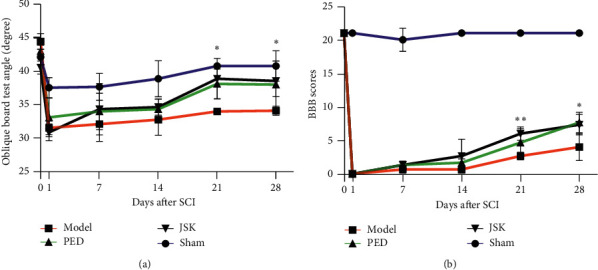
JSK and PED promoted the recovery of motor function in rats with SCI. (a) The oblique board test showed that the motor ability of rats decreased after SCI, and the recovery of motor function could be accelerated after using JSK and PED. At the third week, the promoting effect of JSK was significantly different from the model group. (b) BBB score showed that the motor ability of rats decreased after SCI, and the recovery of motor function could be accelerated after using JSK and PED. At the third and fourth weeks, the promoting effect of JSK was significantly different from that of the model group. The data were presented as mean ± SD; *n* = 3/group, ^∗^*P* < 0.05, and ^∗∗^*P* < 0.01.

The BBB score and oblique board test were carried out before operation and on the 1st, 7th, 14th, 21^st^, and 28th days after operation. The rats with SCI showed a gradual recovery of motor function, while JSK could gradually accelerate the process, and the effect was similar to the PED (Figure 1). The above results show that JSK has the potential to treat SCI.

### 3.2. JSK Can Improve the Microstructure of Neurons in Rats with SCI

**Figure 2:**
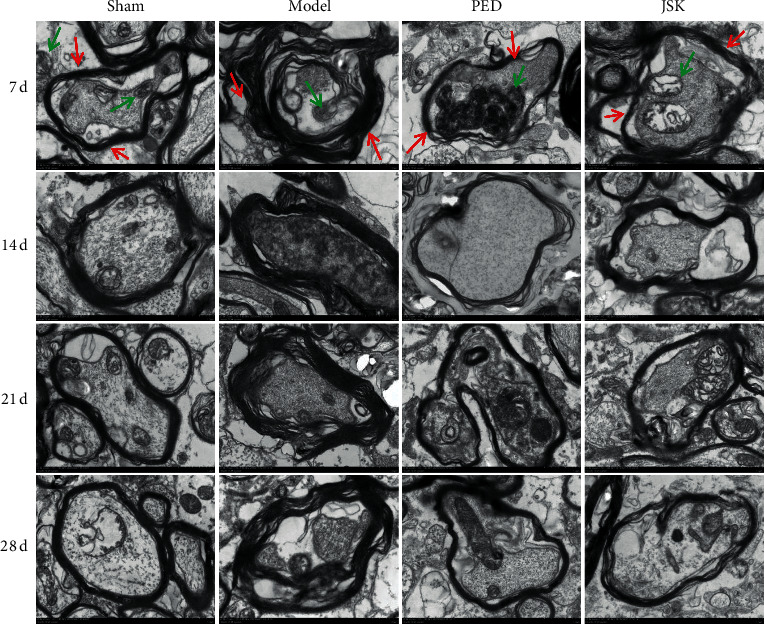
JSK improved the microstructure of neurons in rats with SCI. In the sham group, the shape of spinal myelin sheath was regular, like concentric circular arrangement, and it had a complete structure. The neurons were oval and had complete cell membrane structure, abundant organelles, more mitochondria and rough endoplasmic reticulum, no obvious rupture, or deletion of mitochondrial crest, and the morphology at 7 d–28 d was basically the same. In the model group, the shape of spinal myelin sheath was irregular, and the structure was disordered and incomplete, with partial breakage or deletion, local curl, and wrinkle. The number of neurons and mitochondria decreased, and there were dissolution of part of mitochondria, rupture and deletion of mitochondrial crest, and little change in morphology on 7 d–28 d. The myelin sheath shape of the spinal cord in the JSK group and the prednisone group was improved, the shape was more regular, the structure was more complete, the number of neurons and mitochondria was more, and the morphology of 7 d–28 d was greatly improved compared with the model group. *Note.* The red arrow indicates the myelin sheath and the green arrow indicates the mitochondria.

**Figure 3:**
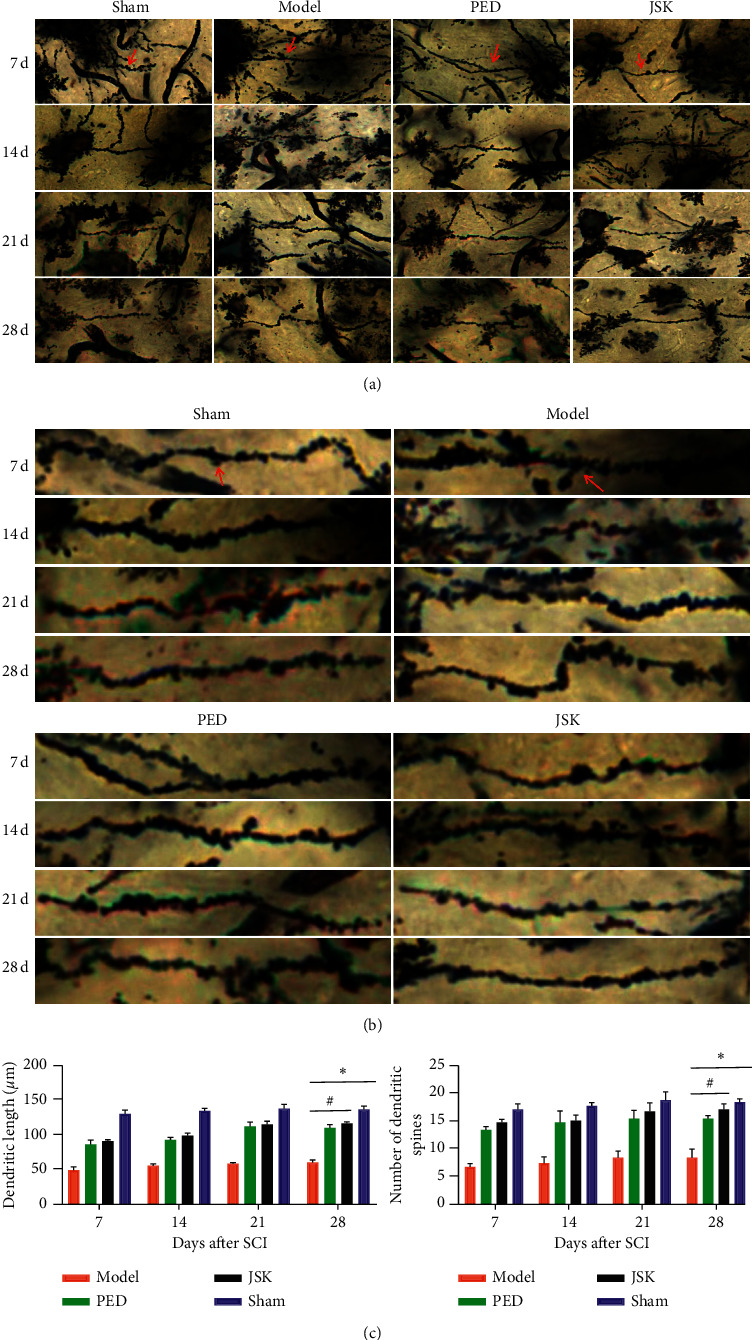
JSK improved the dendritic morphology of neurons in rats with SCI. (a) In the sham group, the morphology of spinal cord dendrites was regular, complete, and long, the dendritic spines arranged neatly, compact, and numerous, and the morphology at 7 d–28 d was basically the same. In the model group, the morphology of spinal cord dendrites was irregular, partially missing, and short, and the arrangement of dendritic spines was disordered and sparse, the number was less, and the morphological changes of 7 d–28 d were not much. In prednisone group and JSK group, the morphology of the spinal cord dendrites was improved, the shape was more regular, the structure was more complete, the length was longer, the dendritic spines were arranged neatly, the density was more uniform, the number was more, and the morphology of 7 d–28 d gradually improved. ((b), (c)) The length of dendrites and the number of dendritic spines in each group were statistically analyzed. The length of dendrites in the sham group was the longest and the number of dendritic spines was the most, while that in the model group was the shortest and the number of dendritic spines was the fewest. Compared with the sham group, the dendritic length and the number of dendritic spines in the model group were significantly decreased, and the dendritic length and the number of dendritic spines in the JSK group were significantly increased compared with the model group. Note. The red arrow indicates the dendritic spine, and the data were presented as mean ± SD; *n* = 3/group, ^∗^*P* < 0.05, and ^#^*P* < 0.05.

The results of the transmission electron microscope showed that the microstructure of spinal myelin sheath and neurons were destroyed after SCI. JSK could alleviate the injury of myelin sheath and neurons after SCI, and at 7 d–28 d, it gradually improved (Figure 2). Golgi staining results showed that, after SCI, the dendrites were destroyed, the length became shorter, and the number of dendritic spines decreased. JSK could alleviate the injury of dendrites and dendritic spines after SCI, and the morphology of 7 d–28 d was gradually improved (Figure 3). The above results suggested that JSK could improve the microstructure of neurons (mitochondria, endoplasmic reticulum, dendritic spines, etc.) in rats with SCI.

### 3.3. JSK Can Reduce the Apoptosis Rate of Neurons in Rats with SCI

**Figure 4:**
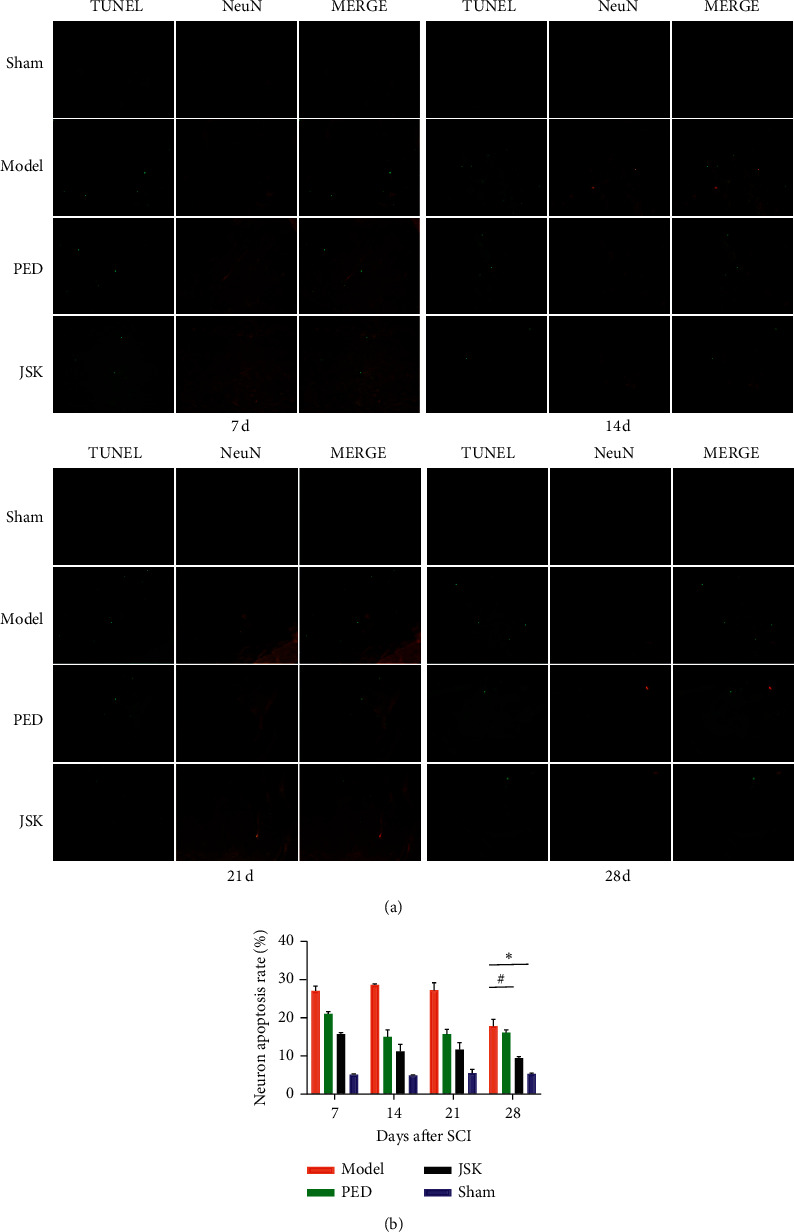
JSK could reduce the apoptosis rate of neurons in rats with SCI. (a) Representative images of Tunel and NeuN double-labeling results. (b) The results of statistical analysis showed that the apoptosis rate of neurons increased after SCI in rats but decreased after intervention of JSK. Compared with the sham group, the apoptosis rate of neurons in the model group increased significantly. Compared with the model group, JSK could significantly reduce the apoptosis rate of neurons. The data were presented as mean ± SD; *n* = 3/group, ^∗^*P* < 0.05, and ^#^*P* < 0.05.

The double-labeling results of TUNEL and NeuN showed that the apoptosis rate of neurons increased after SCI, while JSK could reduce the apoptosis rate of neurons after SCI, and gradually improved between 7 d–28 d (Figure 4).

### 3.4. JSK Can Inhibit the Expression of NgR in Neurons of Rats with SCI

**Figure 5:**
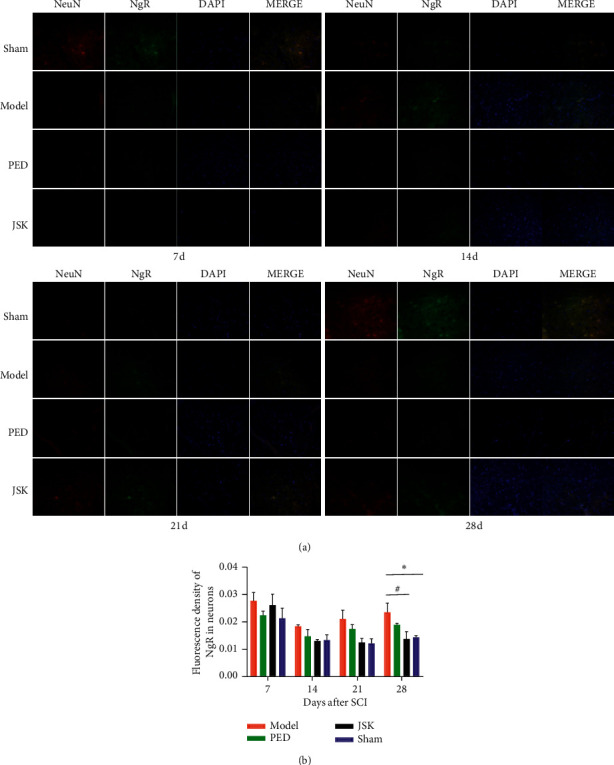
JSK could reduce the expression of NgR in neurons of rats with SCI. (a) Representative images of double-labeling results of NeuN and NgR. (b) The results of statistical analysis showed that the expression of NgR in neurons increased after SCI in rats but decreased after intervention of JSK. Compared with the sham group, the expression of NgR in the model group was significantly increased, and compared with the model group, JSK could significantly reduce the expression of NgR in neurons. The data were presented as mean ± SD; *n* = 3/group, ^∗^*P* < 0.05, and ^#^*P* < 0.05.

The double-labeling results of NeuN and NgR showed that the expression of NgR in neurons increased after SCI, but JSK could reduce the expression of NgR in neurons after SCI. On the 28th day, JSK significantly inhibited the expression of NgR in neurons (Figure 5).

### 3.5. JSK Can Inhibit the Expression of NgR/RhoA/ROCK in the Injured Area of Rats with SCI

**Figure 6:**
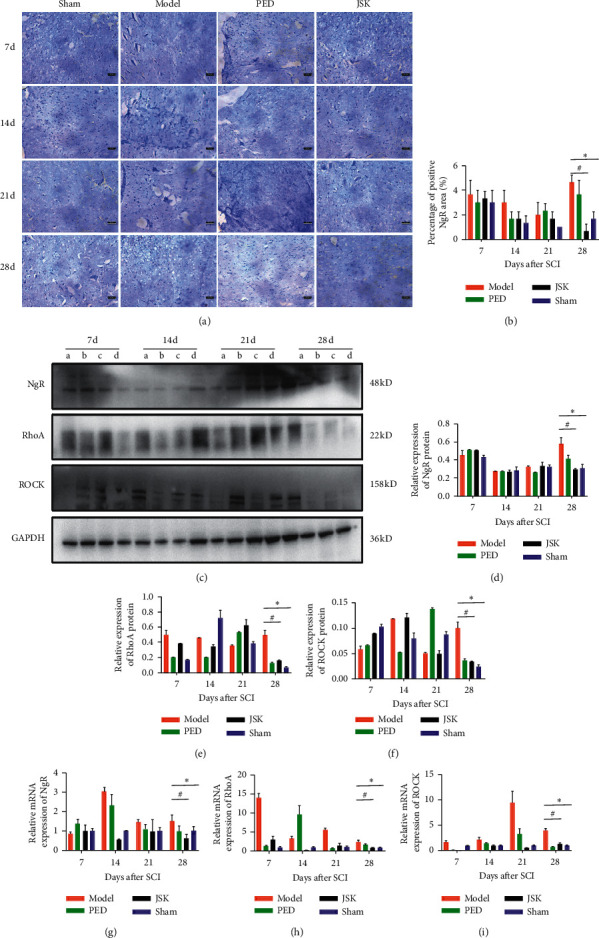
JSK could reduce the expression of NgR in the injured area of rats with SCI. (a) Representative images of immunohistochemistry. (b) Immunohistochemical statistics showed that, on the 28th day, the expression of NgR in the injured area of spinal cord increased but decreased after intervention of JSK. Compared with the sham group, the expression of NgR in the model group was significantly increased, and compared with the model group, JSK could significantly reduce the expression of NgR. (c) Representative images of western blot result. ((d), (e), (f), (g), (h), and (i)) The statistical results of western blot and qRT-PCR showed that, on the 28th day, the expression of NgR/RhoA/ROCK in the injured area of spinal cord increased but decreased after intervention of JSK. Compared with the sham group, the expression of NgR/RhoA/ROCK in the model group was significantly increased, and compared with the model group, JSK could significantly reduce the expression of NgR/RhoA/ROCK. NOTE: a is the model group, b is the prednisone group, c is the prednisone group, and d is the sham group. The data were presented as mean ± SD; *n* = 3/group, ^∗^*P* < 0.05, and ^#^*P* < 0.05.

Immunohistochemistry showed that the expression of NgR in the injured area of rats increased after SCI, but JSK could reduce the expression of NgR in the injured area of rats with SCI. Western blot and qRT-PCR showed that the expression of NgR/RhoA/ROCK in the injured area of rats increased after SCI, but JSK could reduce the expression of NgR/RhoA/ROCK in the injured area of rats with SCI. On the 28th day, JSK significantly inhibited the expression of NgR/RhoA/ROCK in the injured area of the spinal cord (Figure 6).

## 4. Discussion

It was estimated that the incidence of SCI in the United States was about 54 new cases per million people per year. Although the average life expectancy of patients with SCI has increased greatly in the past few decades, due to the aging of the population, the number of cases may continue to increase, and its serious complications can lead to higher economic burden [34]. However, there are few effective treatments for SCI, and the main treatment is still methylprednisolone, which only slightly improves the clinical results. Therefore, there is an urgent need to find a more effective method for the treatment of SCI. SCI belongs to central nervous system injury, and its self-repair ability is very poor, but it maintains the ability of nerve tissue remodeling and axonal plasticity. Its endogenous repair mechanism is mainly related to the properties of the neurons and glial cells, in particular, the local extracellular environment, and the specific inflammatory process caused by the lesion [34]. The current research direction of SCI mainly includes neutralization of myelin-derived inhibitors (e.g., anti-Nogo-antibodies [35]), downstream inhibition of related intracellular signaling pathways (Rho-GTPase signaling [36]), degradation of glial scar inhibitors (CSPG degradation by enzyme [37–39]), neuronal signal pathway (mTOR/PTEN [40]), and transplantation of neural progenitor cells and stem cells [41].

NgR is a GPI-anchored protein expressed in the neurons and axons of the central nervous system [31, 42]. It mediates the inhibitory effect of three kinds of MAIs by forming a receptor complex with p75 and MAIs [43]. When NgR specifically binds to MAIs, it is transmitted to RhoA by p75 and then acts on the effector ROCK to complete the signal transmission in the cell body, resulting in the collapse of the growth cone and inhibiting axonal regeneration [44–46]. The activation of RhoA is considered to be a key step for MAIs to exert its inhibitory effect on axonal regeneration. Intervention of RhoA inactivating agent C3 transferase in vivo weakened the inhibitory effect of MAIs on axonal growth [47]. In addition, treatment with Y27632, a competitive antagonist of ROCK synthesis of ATP, could still promote neuronal axonal growth in the presence of MAIs [48, 49]. The NgR/RhoA/ROCK signal pathway is an important way to inhibit neuronal axonal regeneration, and NgR is the key to this pathway. The development of the NgR/RhoA/ROCK signal pathway in different periods after SCI should be studied.

**Figure 7:**
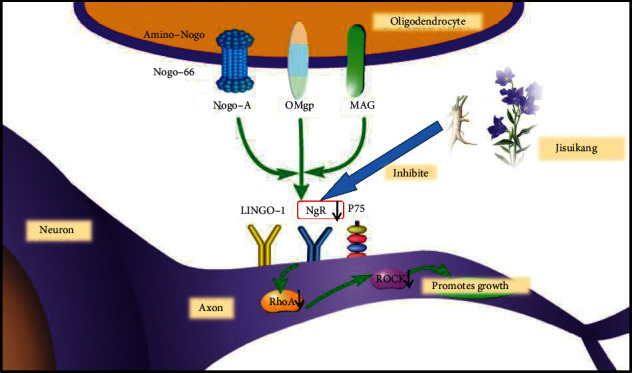
Jisuikang, a Chinese herbal formula, promotes the repair of spinal cord injury in rats by regulating the NgR/RhoA/ROCK signal pathway.

In this experiment, the motor function and the expression of NgR/RhoA/ROCK in rats with SCI were studied. Compared with the model group on the 28th day, the BBB score and oblique board test showed that JSK could promote the recovery of motor function in rats with SCI, Golgi staining, and transmission electron microscope which suggested that JSK could improve the microstructure of neurons (mitochondria, endoplasmic reticulum, dendritic spine, etc.) in rats with SCI. The double labeling of TUNEL and NeuN showed that JSK could reduce the apoptosis rate of neurons in rats with SCI, the double-labeling method of NeuN and NgR showed that JSK could inhibit the expression of NgR in neurons of rats with SCI; immunohistochemistry, western blot, and qRT-PCR all showed that JSK could inhibit the expression of NgR/RhoA/ROCK in the injured area of SCI rat (Figure 7).

In summary, the expression of NgR in the injured area of the spinal cord increased significantly and remained at a high level on the 7th day after SCI. However, on the 28th day, the expression of NgR/RhoA/ROCK in the PED group and JSK group was significantly lower than that in the model group, and JSK could significantly inhibit the expression of NgR/RhoA/ROCK in the injured area to promote nerve regeneration. This result is basically consistent with the results of Guo et al. [14, 50]. JSK can effectively inhibit the expression of NgR in the area of SCI, block the effect of myelin-derived nerve regeneration inhibitor and RhoA/ROCK, improve the microenvironment of axon regeneration, and promote the repair of SCI. In addition, it is worth noting that the motor function of rats with SCI basically recovered continuously before 28 days, and JSK could accelerate this process. The expression of NgR decreased to a certain extent, but the middle part of the results showed that the expression of NgR increased abruptly, which may be due to the fact that NgR is not only the receptor of Nogo-A but also the receptor of MAG and OMgp, while MAG and OMgp may be activated with Nogo-A, thus increasing the expression of NgR. At the same time, the expression of NgR may be increased due to the regulation of other neuronal signal pathways, so the mechanism of multipathway needs to be further studied.

## Data Availability

The data sets used and/or analysed during the current study are available from the corresponding author on reasonable request.
